# Treatment of HEV Infection in Patients with a Solid-Organ Transplant and Chronic Hepatitis

**DOI:** 10.3390/v8080222

**Published:** 2016-08-15

**Authors:** Nassim Kamar, Sébastien Lhomme, Florence Abravanel, Olivier Marion, Jean-Marie Peron, Laurent Alric, Jacques Izopet

**Affiliations:** 1 Department of Nephrology and Organ Transplantation, CHU Rangueil, Toulouse 31059, France; marion-olivier@hotmail.fr; 2 INSERM U1043, IFR–BMT, CHU Purpan, Toulouse 31000, France; lhomme.s@chu-toulouse.fr (S.L.); abravanel.f@chu-toulouse.fr (F.A.); izopet.j@chu-toulouse.fr (J.I.); 3 University Toulouse III—Paul Sabatier, Toulouse 31000, France; peron.jm@chu-toulouse.fr (J.-M.P.); alric.l@chu-toulouse.fr (L.A.); 4 Laboratory of Virology, CHU Purpan, Toulouse 31000, France; 5 Department of Hepatology, CHU Purpan, Toulouse 31000, France; 6 MR 152 IRD-Toulouse III University, Toulouse 31000, France; 7 Internal Medicine-Digestive Department, CHU Purpan, Toulouse, 31000, France

**Keywords:** hepatitis E, ribavirin, immunosuppressants, interferon, sustained virological response

## Abstract

Hepatitis E virus (HEV) infection can cause hepatic and extra-hepatic manifestations. Treatment of HEV infection has been thoroughly studied in solid-organ-transplant patients who have developed a chronic HEV infection. In this review, we report on our current knowledge regarding treatment of HEV infection.

## 1. Introduction

It is known that hepatitis E virus infections with genotypes 1 and 2 (HEV-1 and -2) are responsible for self-limiting hepatitis, and for fulminant hepatitis in patients with underlying chronic liver disease and in pregnant women [1]. However, no cases of chronic hepatitis have been described in these settings [1]. Conversely, HEV genotypes 3 and 4 (HEV-3 and -4) mainly induce self-limiting hepatitis, although also fulminant hepatitis in patients with chronic liver disease, but not in pregnant women [1]. HEV-3 and -4 can also cause chronic infection in patients receiving immunosuppressive agents [2]. Indeed, the first cases of chronic hepatitis were described in patients with a solid-organ transplant (SOT) [2]. Thereafter, several case series have reported on chronic HEV infection in patients that have undergone stem-cell transplantation [3], hematology patients that have received chemotherapy [4], rheumatology patients that have received monoclonal antibodies [5], and patients infected by human immunodeficiency virus (HIV) that have low CD4 counts [6].

The impact of chronic HEV infection has been mainly evaluated in SOT patients. Rapid progression of liver fibrosis and up to a 10% incidence of cirrhosis can develop within a relatively short period after infection [7]. In addition, all HEV genotypes are associated with extra-hepatic manifestations: i.e., mainly neurological disorders and HEV-induced glomerulonephritis [8,9,10]. Hence, hepatic and extra-hepatic manifestations have prompted clinicians to propose appropriate treatments for HEV infection. Treatments for HEV infection were initially evaluated in SOT patients. In this review, we describe our current knowledge regarding treatment of HEV infection within different settings.

## 2. Reducing Immunosuppressive Therapy

Chronic HEV infection was first described in SOT patients infected by HEV-3 and thereafter in those infected by HEV-4. In vivo, low CD4 and CD8 counts at the time of HEV infection, a shorter time since transplantation, and a shorter time from an acute rejection episode have been associated with evolution to chronicity [7,11]. The use of tacrolimus (rather than cyclosporine A), which is a potent immunosuppressive drug, has been identified as an independent predictive factor for chronic hepatitis [7]. In addition, a lower T-cell response and inflammatory response have been observed in transplant patients with chronic hepatitis [12,13]. All these data suggest that patients that are heavily immunosuppressed have a high risk of developing a chronic infection. In vitro data have shown that the majority of immunosuppressive drugs, i.e., cyclosporine A, tacrolimus, sirolimus, and everolimus, increase HEV replication [14]. Only mycophenolic acid can decrease HEV replication in vitro [14].

In a retrospective multicenter study, it was observed that HEV clearance occurred in 30% SOT patients with chronic HEV infection after reducing immunosuppressive therapies that principally targeted T-cells [7]. Indeed, patients who were cleared of the virus achieved a lower tacrolimus trough level and needed lower daily doses of steroids compared to those who remained viremic [11]. Based on in vitro and in vivo data (when possible) a reduction in immunosuppressive therapy seems to be the first-line therapeutic option as it can increase T-cell response, thus allowing HEV clearance.

## 3. Antiviral Therapies

Despite a reduction in immunosuppressive therapy, two-thirds of patients remain viremic, and because immunosuppressants cannot be reduced in all transplant-patients, an effective antiviral therapy is needed.

### 3.1. Pegylated Interferon

In vitro, interferon had a moderate antiviral activity [15,16]. In vivo, pegylated interferon was given to a few patients, mostly for a period of three months, except for one patient who received it for 12 months. The sustained virological response (SVR) at six months was 100% [17,18,19]. However, interferon cannot be used safely in patients who have received a heart, lung, pancreas or kidney allograft because it increases the risk of acute rejection due to its immunostimulatory effect [20].

### 3.2. Ribavirin

Several research groups have tried using ribavirin alone in SOT patients with chronic hepatitis. Unexpectedly, the results have been impressive [21,22,23].

In a retrospective multicenter French study, the effect of ribavirin alone was assessed in 59 SOT patients with chronic HEV infection [24]. At the initiation of therapy, the median daily dose of ribavirin was 8.1 (0.6–16.3) mg/kg. Among the 59 patients, 17 had been given ribavirin at 600 mg/day and 17 others had received ribavirin at a dose of 800 mg/day. Ribavirin dose reduction was required in 30% of cases because of ribavirin-induced anemia and two patients had to stop for 10 and 15 days because of severe anemia. The duration of therapy ranged from one to 18 months. However, the large majority were treated for three months (*n* = 36). The SVR at six months was 78% (*n* = 46) [24]. Two patients were lost to follow-up, another patient was initially a null responder and 10 other patients relapsed after ribavirin therapy was stopped. The null responder was retreated and cleared the virus. Six of the 10 relapsers who were initially treated for three months were retreated for six months. SVR was then observed in four of these patients and by the time the paper was published, the two remaining patients were also cleared of the virus. However, the follow-up was not sufficiently long to evaluate if these patients achieved a SVR or not. Hence, the overall SVR was 85% [24]. No statistically significant difference was observed between patients who were treated for three months or less and those who received ribavirin for more than three months. As expected, anemia was the main adverse event observed, and up to 40% of these patients required recombinant erythropoietin [24]. Hence, ribavirin as a monotherapy has become the treatment of choice for chronic HEV infection.

The mechanism of action of ribavirin on HEV remains unclear. It has been suggested that ribavirin inhibits HEV replication through depletion of guanosine triphosphate (GTP) [25]. As mentioned above, mycophenolic acid, an inhibitor of inosine-5′-monophosphate dehydrogenase (which decreases GTP production), was found to decrease HEV replication in vitro [14]. It has also been observed that ribavirin and mycophenolic acid have a synergistic anti-HEV effect in vitro [14]. In vivo, no difference in the slope of HEV RNA concentration and the number of SVRs was observed between SOT patients who received ribavirin either with or without mycophenolic acid [26]. It has been recently shown that ribavirin increases viral heterogeneity and that ribavirin-induced mutagenesis seems to be reversible after therapy is stopped [27].

Cases of failed ribavirin treatment have been reported in patients infected with an HEV variant that has the G1634R mutation in the viral polymerase [28]. This mutation does not provide ribavirin resistance in vitro, but increases the replicative capacity of HEV [28]. In a series of 63 SOT patients with chronic infection, G1634R variants were detectable in 23 patients (36.5%) before therapy [29]. The median proportion of G1634R variants within the quasispecies was 8.2% (1.3%–100%). The presence of the G1634R mutation before treatment did not have any impact on the early virological response, on the SVR, or on virological response after retreatment [29]. G1634R variants were detectable before therapy in 31% and 47.5% of patients with and without a SVR (*p* = 0.2) [29]. In relapsers, Todt et al. found that G1634R variants were also detected under therapy [27]. Recently, it has been shown that other dominant mutations can occur in the polymerase region, i.e., K1383N, Y1587F, D1384G, V1479I, and K1398R [16]. These mutations also increase the replicative capacity of HEV [30]. However, interestingly, they also improve the antiviral activity of ribavirin [30].

### 3.3. Predictive Factors for a SVR in Patients Receiving Ribavirin

A lower lymphocyte count at the initiation of ribavirin therapy has been associated with more relapses after ribavirin is stopped [24]. Persistent HEV shedding in the stools at the end of therapy leads to more HEV-replication relapses after the end of therapy [31]. A decrease in HEV RNA concentration of more than 0.5 log copies/mL within the first week after initiating ribavirin therapy was found to be a predictive factor for a SVR [26]. Interestingly, ribavirin trough levels at one week and at steady state, i.e., at 2 months, did not impact on virological response or the SVR [26].

### 3.4. Alternative Antiviral Therapy

In vitro, it has been shown that sofosbuvir may inhibit HEV replication and may increase the antiviral effect when combined with ribavirin [32]. However, its potential effect on HEV replication in vivo has not been established.

## 4. Treatment of HEV Infection with Additional Complications

### 4.1. Treatment in Hematology Patients

Few case reports and case series have been published on the treatment of hematological patients, i.e., patients receiving chemotherapy and/or stem-cell-transplant recipients. Similar to SOT patients, hematology patients have been successfully treated with pegylated interferon or ribavirin [33,34,35]. Nine out of 12 stem-cell-transplant recipients treated with ribavirin achieved a SVR [35].

### 4.2. Treatment of HEV Infection in HIV-Positive Patients

Very few cases of chronic HEV infection have been reported in HIV patients. However, those who have been treated with pegylated interferon, ribavirin, or both, have achieved a SVR [36,37,38,39].

### 4.3. Treatment of Acute-Phase HEV

A few patients who have presented with severe acute HEV infection, or acute or chronic hepatitis, have been given ribavirin [40,41]. A rapid decrease in HEV RNA concentration has been obtained and HEV clearance achieved. Nevertheless, no trial so far has compared patients that have received or not received ribavirin in this setting.

### 4.4. Treatment of HEV-Induced Extra-Hepatic Manifestations

Antiviral therapy was used in patients presenting HEV-induced neurological symptoms such as Guillain Barré Syndrome [42]. Although it allowed clearing of HEV, its effects on the outcome of neurological symptoms is unknown. In contrast, antiviral HEV therapy was efficient in treating patients who developed HEV-associated kidney injuries [43,44].

## 5. Suggested HEV Infection Therapy Algorithm

**Figure 1:**
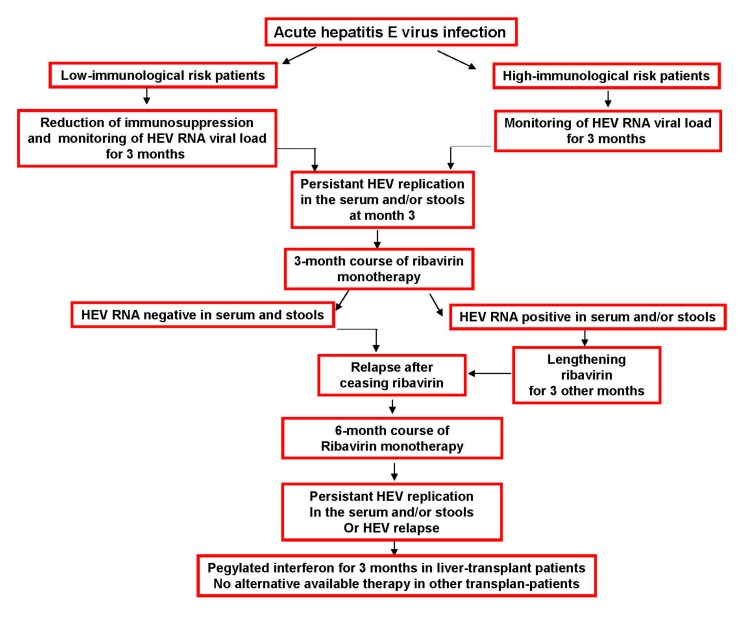
Treatment of hepatitis E virus persistent infection in solid-organ-transplant patients.

Although there is no high level evidence at different stages, we suggest the following practical management algorithm for treating HEV infection in transplant patients with chronic hepatitis. In patients with a low immunological risk it is reasonable to decrease immunosuppression, especially calcineurin inhibitors, as soon as HEV infection is diagnosed and to then wait for three months. If HEV infection is not cleared within three months after diagnosis, an antiviral therapy can be proposed. In patients with a high immunological risk, immunosuppression cannot usually be reduced. Thus, waiting for three months before proposing antiviral therapy is suggested (Figure 1).

In all transplant patients, ribavirin therapy is the treatment of choice. The recommended dose is unknown. Nevertheless, in patients with impaired kidney function, ribavirin dose should be adapted to kidney function [45]. In most studies, patients were given 600 to 800 mg/day [24]. Three months after initiating ribavirin therapy, if HEV RNA is negative in both the sera and stools, ribavirin can be stopped. If HEV RNA remains positive in the stools after three months, even if it is negative in the sera, ribavirin therapy should be prolonged for an additional three months. If HEV viremia increases after ceasing ribavirin therapy, a longer course of ribavirin, i.e., 6 months, can be proposed. In patients who present with a relapse under ribavirin or who are resistant to ribavirin, there is no alternative, except for liver-transplant patients for whom pegylated interferon can be proposed.

## 6. Conclusions

Ribavirin is the first-choice therapy to treat HEV infection. Further studies are needed to identify novel antiviral therapies for patients that are resistant or partial responders to ribavirin.
